# Influenza A Virus Infections in Dromedary Camels, Nigeria and Ethiopia, 2015–2017

**DOI:** 10.3201/eid2601.191165

**Published:** 2020-01

**Authors:** Daniel K.W. Chu, Ranawaka A.P.M. Perera, Abraham Ali, Jamiu O. Oladipo, Gezahegne Mamo, Ray T.Y. So, Ziqi Zhou, Yen Yeen Chor, Chak Kai Chan, Desalegn Belay, Adamu Tayachew, Mesfin Mengesha, Feyesa Regassa, Nga Ting Lam, Leo L.M. Poon, Malik Peiris

**Affiliations:** The University of Hong Kong, Hong Kong, China (D.K.W. Chu, R.A.P.M. Perera, J.O. Oladipo, R.T.Y. So, Z. Zhou, Y.Y. Chor, C.K. Chan, N.T. Lam, L.L.M. Poon, M. Peiris);; Ethiopian Public Health Institute, Addis Ababa, Ethiopia (A. Ali, D. Belay, A. Tayachew, M. Mengesha, F. Regassa);; Addis Ababa University, Bishoftu, Ethiopia (G. Mamo)

**Keywords:** influenza A, dromedary camels, camels, reverse zoonosis, Nigeria, Ethiopia, influenza A virus, respiratory infections, viruses, H1N1, influenza A(H1N1)pdm09, H3N2, zoonoses, phylogenetic analysis, serology, influenza

## Abstract

We examined nasal swabs and serum samples acquired from dromedary camels in Nigeria and Ethiopia during 2015–2017 for evidence of influenza virus infection. We detected antibodies against influenza A(H1N1) and A(H3N2) viruses and isolated an influenza A(H1N1)pdm09–like virus from a camel in Nigeria. Influenza surveillance in dromedary camels is needed.

Aquatic wild birds are the natural reservoir of influenza A virus, which comprises 16 hemagglutinin and 9 neuraminidase subtypes. Influenza A virus subtypes H1N1, H2N2, and H3N2 have caused pandemics in humans, and subtypes H1N1 and H3N2 circulate in humans as seasonal influenza. Pandemic influenza arises when an animal influenza virus evolves through the reassortment of animal and human virus gene segments (antigenic shift) to sustainably transmit in humans. Avian and swine influenza viruses have caused zoonotic infections, some resulting in fatal disease. Thus, influenza virus surveillance in animals is needed for pandemic preparedness (*1*).

Dromedary camel populations, estimated to be 30 million globally, can be found in parts of Africa, the Middle East, and Central Asia, often in close proximity to humans. An equine influenza A(H3N8) virus (*2*) and human influenza A/USSR/90/77(H1N1)–like viruses (which were associated with fatal disease in 1980–1983) (*3*) have been isolated from Bactrian camels in Mongolia. However, little is known of influenza A virus infections in dromedary populations. Therefore, we carried out a study to determine the prevalence of influenza A virus infection in dromedary camels.

As part of an investigation of Middle East respiratory syndrome coronavirus conducted during October 2015–February 2016 (*4*), we collected 2,166 nasal swabs and 150 serum samples from dromedaries at an abattoir in Kano, Nigeria. We also collected 102 nasal swabs and 100 serum samples from nomadic dromedary herds in Amibara, Ethiopia (in January 2017); 109 nasal swabs from herds in Asayita, Ethiopia (in July 2017); and 83 nasal swabs from herds in Dubti, Ethiopia (in July 2017). We put nasal swabs in virus transport medium, stored samples frozen at −80°C, and shipped them to Hong Kong, China, on dry ice for laboratory tests. We performed RNA extraction and detected the influenza A virus matrix gene using molecular methods (Appendix) (*5*).

Twelve nasal swabs from camels in Nigeria collected at different times (October [n = 5] and November [n = 5] 2015 and January [n = 1] and February [n = 1] of 2016) were positive for the influenza A virus matrix gene; cycle thresholds for these samples were 33.7–38.9 (Appendix Table). One of the nasal swabs collected in Amibara was also positive (cycle threshold 37.8). We inoculated quantitative reverse transcription PCR–positive samples onto Madin Darby canine kidney cells grown in 24-well plates in minimum essential medium with tosylsulfonyl phenylalanyl chloromethyl ketone–treated trypsin (2 µg/mL) (Appendix) (*6*). We examined inoculated cells for cytopathic effect and passaged each culture twice. We defined virus detection as the appearance of cytopathic effect or the hemagglutination of turkey erythrocytes. We were able to isolate 1 virus, which we designated A/dromedary/NV1337/2016 (H1N1), from a swab collected in Nigeria on January 22, 2016 (Appendix Table).

We performed full-genome sequencing of A/dromedary/NV1337/2016 (Appendix) as previously described (*6*). We achieved sequencing read coverages of >100 for each nucleotide and were able to deduce the full virus genome (GenBank accession nos. MN453859–66). All 8 gene segments showed their highest identity (>99.8) to contemporary influenza A(H1N1)pdm09 viruses (data not shown).

In a phylogenetic analysis, we compared the hemagglutinin gene of A/dromedary/NV1337/2016 with that of other influenza A(H1N1)pdm09 viruses available from GenBank and GISAID (https://platform.gisaid.org) (Figure). The dromedary influenza A virus isolated in Nigeria in January 2016 was similar to other influenza viruses circulating in humans at the same time. The sampling dates for the influenza viruses detected in camels in Nigeria overlapped with the human influenza virus season, which typically occurs during October–March (Appendix Table) (*7*). This finding suggests reverse zoonosis of influenza viruses from humans to dromedaries. Whether these viruses were subsequently maintained in dromedary populations via camel-to-camel transmission is not clear. Further studies are needed to address this question. Transmission of influenza A virus also occurs from humans to swine, and these viruses can be maintained in swine populations for variable periods, sometimes decades (*8*).

**Figure:**
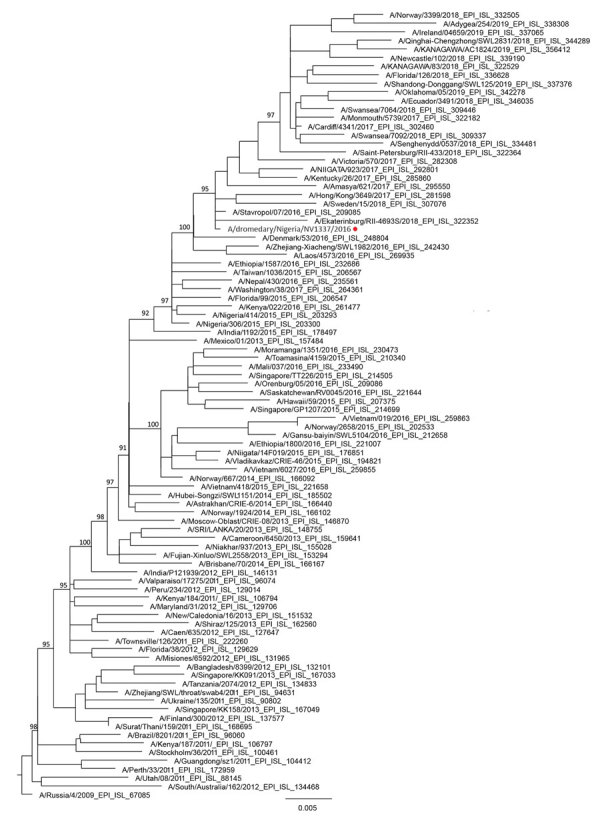
Maximum-likelihood phylogenetic tree showing relationship of influenza A(H1N1)pdm09 virus from dromedary camel, Nigeria, January 2016 (red circle), relative to other influenza A(H1N1)pdm09 viruses from humans worldwide on the basis of the hemagglutinin gene. Tree was constructed by using a general time-reversible model with FastTree (https://www.geneious.com/plugins/fasttree-plugin) and PhyML (http://www.atgc-montpellier.fr/phyml) (Appendix). Tree is rooted with an influenza A(H1N1)pdm09 virus collected in 2009. Bootstrap support values for the major branches are shown. Scale bar indicates number of nucleotide changes per base pair.

We tested serum samples from dromedary camels for hemagglutination inhibition (HI) antibody against A/dromedary/NV1337/2016(H1N1) using standard methods (Appendix) (*5*); 4 serum samples from camels in Amibara had HI antibody (titers 1:40, 1:80, 1:160, and 1:160). The dromedaries that had influenza A virus RNA–positive nasal swabs were negative for HI antibody. Dromedaries recently infected with a virus are expected to be seronegative for that virus because antibody responses against viruses take around a week to develop (*9*), by which time nasal swab specimens are often negative for that virus’s genomic material. Microneutralization tests are more appropriate for testing antibody to contemporary H3N2 viruses (*10*); hence, we also tested serum samples for antibody to A/Hong Kong/4801/2014(H3N2) virus using the microneutralization test (Appendix). In total, 1 serum sample from a camel in Nigeria was positive at a titer of 1:80.

In conclusion, we provide evidence of influenza A virus infection in dromedaries. Our findings indicate that influenza virus surveillance in dromedary camel populations is needed.

Appendix More information on influenza A virus infections in dromedary camels, Nigeria and Ethiopia, 2015–2017.
