# Supplementary material for: Influenza A Virus Infections in Dromedary Camels, Nigeria and Ethiopia, 2015–2017
Source: Emerg Infect Dis. 2020 Jan;26(1):173–6. doi: 10.3201/eid2601.191165 (PMC6924894; doi:10.3201/eid2601.191165)
Supplement: Appendix — More information on influenza A virus infections in dromedary camels, Nigeria and Ethiopia, 2015–2017. [file 19-1165-Techapp-s1.pdf]

Article DOI: https://doi.org/10.3201/eid2601.191165

Influenza A Virus Infections in Dromedary Camels, Nigeria and Ethiopia, 2015–2017

Appendix

Methods

Reverse Transcription PCR (RT-PCR) for Detection of Influenza Viruses

RNA extraction from swabs samples or cell culture isolates was carried out by Biomerieux EasyMag or Roche MagNA pure automated extraction systems with protocols recommended by the manufacturers. Extracted RNA was tested by a real time quantitative RT-PCR (RT-qPCR) assay targeting a conserved region in influenza A matrix gene (*1*). A negative control was included for every 10 specimens tested and a positive control included in each RT-qPCR run.

For detection of influenza A virus, 4µL of RNA was amplified in a ViiA 7 Real-Time PCR System (Thermo Fisher Scientific) with a total reaction-mix volume of 20µL in 1x Taqman Fast Virus 1-step master mix (Applied Biosystems) containing 0.5mM of forward primer (5′-CTTCTAACCGAGGTCGAAACGTA–3′), reverse primer (5′-GGTGACAGGATTGGTCTTGTCTTTA–3) and hydrolysis probe (5′-FAM-TCAGGCCCCCTCAAAGCCGAG-BHQ1–3′) for amplification of the matrix gene of influenza A virus. Cycling conditions were as follows: an initial reverse transcription step at 50°C for 5 minutes and initial denaturation at 95°C for 20 seconds, followed by 40 cycles of 95°C for 5 seconds, 60°C for 30 seconds, 72°C for 12 seconds with default ramp rates of the apparatus in fast reaction mode. Reactions with serial dilutions of known copy number control sample were included in each run for monitoring detection sensitivity and estimating gene copy numbers in samples.

Full-Length Influenza A Virus Genome Sequencing

Full-length cDNAs were synthesized using the uni-12 primer followed by amplification of full-length gene segments with primers targeting both ends of the gene segments (*2*). PCR

products were analyzed with 1% agarose gel electrophoresis. Products with expected segment sizes were sequenced using Illumina HiSeq 2500 system with Nextera XT library preparation method (Illumina). The virus genome was deduced by taking the consensus of sequencing raw reads mapped to a selected pdm09H1N1 reference genome. Sequencing read coverages of at least 100 were achieved for each nucleotide.

Phylogenetic Analysis

Phylogenetic tree of hemagglutinin of influenza A H1 pdm09H1N1 lineage viruses by the maximum likelihood method using IQTree with auto substitution model selection (*3*) and ultrafast bootstrap approximation (*4*). The tree was rooted to a pdm09H1N1 virus collected in year 2009. Support values for the major branches were shown. The length of the scale bar denotes the number of nucleotide changes along the horizontal branches of the tree.

Viral Culture

Madin-Darby canine kidney (MDCK) cells were plated in 24-well plates (TPP® tissue culture plates, Sigma-Aldrich) at $1x10^5$ cells per well in minimum essential medium (MEM) (GIBCO, New York) with 10% fetal bovine serum (*2*). When the cells were semi-confluent (usually within 24 hours) the cells were washed three times with serum free MEM with tosylsulfonyl phenylalanyl chloromethyl ketone (TPCK)-treated trypsin (2µg/ml) (Sigma, St. Louis, Missouri). Each well was inoculated with 100ul of the swab supernatant in MEM and incubated for one hour at 37°C in a CO_2 incubator. Each inoculated well was separated by an un-inoculated cell control well to avoid cross-contamination and provide cell controls. The inoculum was replaced with MEM with trypsin (2µg/ml) and incubated for 5 days. The cells were observed for cytopathic effect (CPE) each day. When CPE appeared or on day 5 if no CPE was seen, aliquots of the cell supernatant was tested for hemagglutination with turkey red blood cells in U-bottomed plates. Culture supernatants with CPE or hemagglutination activity were also tested by RT-PCR for influenza matrix (M) gene and serially passaged in MDCK cells to obtain stock virus for aliquoting and freezing at –80°C.

Serology

Hemagglutination Inhibition (HI) Test

The sera were treated with receptor-destroying enzyme (RDE) (Denka Seiken Co Ltd, Tokyo) at 37°C overnight to remove nonspecific inhibitors. Next morning residual RDE was

destroyed by heat inactivation at 56°C for 30 minutes and then diluted with six volumes of 0.9% NaCl solution before carrying out HI tests. Serial 2-fold dilutions of each RDE treated serum was prepared in duplicate in 96-well microtiter plates. Equal volumes (25uL) each serum dilution was mixed with virus antigen with a hemagglutination titer of 8 in each well. After a 1h incubation, 50µL of a 0.5% solution in PBS of washed turkey red blood cells (TRBC) was added to each well and incubated for 30min. The plates were inspected with tilting. The HI titer of each serum was defined as the highest serum dilution that inhibited hemagglutination and formed a clear button of TRBC at the bottom of the well with a tear-drop appearance being seen when the plate was tilted. Positive and negative control sera were included with each set of titrations. Antigen back titrations were done to ensure that the correct HA dose was used in each run (*5*).

Microneutralization Test

The 3 day microneutralization test as described in reference (*6*) was carried out in 96-well microtiter plates using neutralization of virus cytopathogenic effect (CPE) in Madin-Darby Canine Kidney (MDCK) cells and detectable hemagglutination in the culture supernatant as the read-out for evidence of virus replication. Serial serum dilutions in quadruplicate were mixed with 100 tissue culture infectious dose 50 ($TCID_{50}$) of A/Hong Kong/4801/2014 (H3N2) for 1 hour and then the virus-serum mixture was transferred to pre-formed MDCK cell monolayers. One hour after inoculation, serum-virus mixtures were removed and serum free MEM with 2 ug/ml TPCK trypsin was added to each well. The plates were incubated at 37°C in a CO_2 incubator and cytopathic effect was observed to determine the highest serum dilution that neutralized CPE in ≥50% of the wells. On day 3, an aliquot of each culture supernatants were tested for hemagglutination with turkey red blood cells. A virus back titration and positive and negative control sera were included in each assay.

Appendix References

1. Duchamp MB, Casalegno JS, Gillet Y, Frobert E, Bernard E, Escuret V, et al. Pandemic A(H1N1)2009 influenza virus detection by real time RT-PCR: is viral quantification useful? Clin Microbiol Infect. 2010;16:317–21. PubMed https://doi.org/10.1111/j.1469-0691.2010.03169.x

2. Baudon E, Chu DKW, Tung DD, Thi Nga P, Vu Mai Phuong H, Le Khanh Hang N, et al. Swine influenza viruses in Northern Vietnam in 2013–2014. Emerg Microbes Infect. 2018;7:1–16. PubMed https://doi.org/10.1038/s41426-018-0109-y

3. Nguyen LT, Schmidt HA, von Haeseler A, Minh BQ. IQ-TREE: a fast and effective stochastic algorithm for estimating maximum-likelihood phylogenies. Mol Biol Evol. 2015;32:268–74. PubMed https://doi.org/10.1093/molbev/msu300

4. Hoang DT, Chernomor O, von Haeseler A, Minh BQ, Vinh LS. UFBoot2: Improving the Ultrafast Bootstrap Approximation. Mol Biol Evol. 2018;35:518–22. PubMed https://doi.org/10.1093/molbev/msx281

5. Lennette EH, Lennette DA, Lennette ET. Diagnostic procedures for viral, rickettsial, and chlamydial infections. 7th Edition. American Public Health Association, Washington, DC; 1999.

6. Laurie KL, Engelhardt OG, Wood J, Heath A, Katz JM, Peiris M, et al.; CONSISE Laboratory Working Group participants. International Laboratory Comparison of Influenza Microneutralization Assays for A(H1N1)pdm09, A(H3N2), and A(H5N1) Influenza Viruses by CONSISE. Clin Vaccine Immunol. 2015;22:957–64. PubMed https://doi.org/10.1128/CVI.00278-15

Appendix Table. Summary of influenza A M gene RT-qPCR positive samples*

Country	Lab ID	Sampling date, day/month/year	Influenza A M gene Ct	Hemagglutination titer passage 1	Hemagglutination titer passage 2
Nigeria	NV0282	22/10/2015	37.25	neg	neg
Nigeria	NV0325	24/10/2015	37.69	neg	neg
Nigeria	NV0334	24/10/2015	38.30	neg	neg
Nigeria	NV0467	20/10/2015	37.71	neg	neg
Nigeria	NV0480	30/10/2015	36.85	neg	neg
Nigeria	NV0619	04/11/2015	37.56	neg	neg
Nigeria	NV0714	09/11/2015	34.96	neg	neg
Nigeria	NV0823	13/11/2015	33.67	neg	neg
Nigeria	NV0974	19/11/2015	38.83	neg	neg
Nigeria	NV1250	30/11–2015	38.87	neg	neg
Nigeria	NV1337	22/1/2016	37.60	Positive HA titer 32	Positive HA titer 64
Nigeria	NV1875	2/11/2016	37.53	neg	neg
Ethiopia	CAC4377	1/12/2017	37.77	neg	neg

*Ct, cycle threshold; HA, hemagglutination; ID, identification; M, matrix; neg, negative; RT-qPCR, quantitative reverse transcription PCR.
